# Toward a Consensus on Guiding Principles for Health Systems Strengthening

**DOI:** 10.1371/journal.pmed.1000385

**Published:** 2010-12-21

**Authors:** Robert C. Swanson, Annette Bongiovanni, Elizabeth Bradley, Varnee Murugan, Jesper Sundewall, Arvind Betigeri, Frank Nyonator, Adriano Cattaneo, Brandi Harless, Andrey Ostrovsky, Ronald Labonté

**Affiliations:** 1 Department of Health Sciences, Brigham Young University, Provo, Utah, United States of America; 2 The QED Group LLC, Washington, D.C., United States of America; 3 Yale School of Public Health, New Haven, Connecticut, United States of America; 4 Division of Global Health, Karolinska Institute, Stockholm, Sweden; 5 Health Systems Action Network, New Delhi, India; 6 Ministry of Health, Accra, Ghana; 7 Institute for Maternal and Child Health, Trieste, Italy; 8 EntrePaducah, Paducah, Kentucky, United States of America; 9 Health Systems Action Network, Baltimore, Maryland, United States of America; 10 Institute of Population and Department of Epidemiology and Community Medicine, University of Ottawa, Ontario, Canada

## Abstract

Based upon a review of the literature, Robert Chad Swanson and colleagues present a set of guiding principles for health systems strengthening.

**Summary Points**

- Despite the expanding consensus about the need for health systems strengthening (HSS), there is a lack of a common definition and set of guiding principles that can inform strategic frameworks used to develop policy, practice and evaluations.
- Without a set of agreed-upon principles, these frameworks may be unclear and inconsistent, limiting the ability for collective learning, innovation, and improvement.
- A set of ten guiding principles for HSS is proposed in this paper that is based upon a systematic review and consultation with experts in three countries.
- They are: holism, context, social mobilization, collaboration, capacity enhancement, efficiency, evidence-informed action, equity, financial protection, and satisfaction.
- The authors welcome and encourage further discussion of these findings at all levels so that a broad consensus on HSS principles is obtained.

## Introduction

A renewed focus on health systems strengthening (HSS) in global health has emerged in recent years. The World Health Organization (WHO) and others have promoted HSS as essential to attaining the Millennium Development Goals and to improving global health outcomes [1],[2]. This recent increase in interest is highlighted by the organization of the First Global Symposium on Health Systems Research, held in November 2010 [3]. Additionally, numerous funding opportunities with an emphasis on HSS have been established, including a collaborative effort between the Global Alliance for Vaccines and Immunization (GAVI Alliance), The Global Fund to Fight AIDS, Tuberculosis and Malaria (Global Fund), and the World Bank [4], as well as US President Obama's Global Health Initiative [5].

Despite the growing consensus for the need for HSS, there is little agreement on strategies for its implementation [6]. Widely accepted guiding principles could provide a common language for strategy development and communication in the global community. Without a set of agreed-upon principles, frameworks for policy, practice, and evaluation may be unclear, overly narrow, or inconsistent [7], limiting the ability for collective learning, innovation, and improvement. Here we suggest a list of ten guiding principles necessary for effective HSS.

## Methodology for Developing a Set of HSS Principles

We employed several methods for developing a proposed set of guiding principles for HSS.

First, we conducted a systematic review of 633 documents from peer-reviewed and gray literature for HSS definitions, examples, and explanations. For peer-reviewed sources, we searched PubMed, Google Scholar, and Scopus for literature published from 2000 to 2009 using the search terms “health system(s) strengthening.” For gray literature sources, we used Google to identify HSS definitions or approaches in Web sites, conference proceedings, interviews, textbooks, and policy documents. Based on our review of abstracts and summaries, we excluded documents (*n* = 296) that did not meet the following inclusion criteria: contained a definition, explanation, or example of strengthening or improving health systems; were relevant to the low- or middle-income country context; and were available in full text in English. Two researchers then independently conducted a full-text review of the remaining 337 documents in order to categorize HSS definitions by keywords developed inductively during the review process. We summarized the data using a frequency distribution of keywords tagged. A separate researcher then reviewed the 337 documents for HSS guiding principles. (See Text S1 for more details on the methodology of the systematic review, and Text S2 for a list of the keywords identified.)

We also reviewed 11 key publications (Box 1) that address HSS, and we repeatedly consulted more than 30 global health professionals representing different aspects of health systems involvement (see Acknowledgments). Based on our systematic review, extensive consultations, and analysis of the current HSS literature, we identified ten principles for HSS to address the current lack of consensus. Finally, we discussed the principles at six global health conferences in three countries (see Text S3 for a list of conferences). The systematic review established the need for a consensus and assisted in generating an initial set of principles on which there was some normative agreement. Our methods to refine the list of principles involved iterative processes that incorporated not only the evidence from the review, but also the considerable field experiences of those who participated in the process, and rigorous discussion amongst the authors of the paper.

**Box 1. Key Documents That Outline Major Health Systems Strengthening Principles**

- Systems Thinking for Health Systems Strengthening [2]
- Everybody's Business: Strengthening Health Systems to Improve Health Outcomes [9]
- GAVI Alliance Health Systems Strengthening Guiding Principles [13]
- Just and Lasting Change: When Communities Own Their Futures [24]
- Getting Health Reform Right: A Guide to Improving Performance and Equity [27]
- The World Health Report 2000 – Health Systems: Improving Performance [30]
- The Paris Declaration on Aid Effectiveness [31]
- Challenging Inequity through Health Systems. Final Report, Knowledge Network on Health Systems 2007 [36]
- Declaration of Alma-Ata [40]
- The NGO Code of Conduct for Health Systems Strengthening [41]
- Health Systems and the Right To Health: An Assessment of 194 Countries [42]

Although our proposed principles are applicable to diverse geographical, sociocultural, and socioeconomic settings, we focused on health systems in low- and middle-income countries from the perspective of external funders and stakeholders who play a role in developing strategic frameworks for policy, practice, or evaluations. We use the WHO definition of a health system as a network that “comprises all organizations, institutions and resources devoted to producing actions whose primary intent is to improve health” [8].

## Outcomes of the Review and Discussions

### HSS Definitions and Approaches in the Literature

Review of the 337 documents identified 39 distinct keywords that characterized HSS (see Text S2 for a complete list). The most commonly used keywords were “health workforce related national policies and investment plans; norms, standards and training” (in 43.8% of all documents) and “health service delivery and packages/delivery models; infrastructure; demand for care” (in 43% of all documents). Although keywords relating to the WHO's six health systems building blocks (Box 2) [9] were the most commonly used, the mention of all six building blocks occurred in only 5.6% of all documents.

**Box 2. The WHO Health Systems Building Blocks [9]**

1. **Service delivery:** Packages; delivery models; infrastructure; management; safety and quality; demand for care

2. **Health workforce:** National workforce policies and investment plans; advocacy; norms, standards, and data

3. **Information:** Facility and population-based information and surveillance systems; global standards, tools

4. **Medical products, vaccines, and technologies:** Norms, standards, policies; reliable procurement; equitable access; quality

5. **Financing:** National health financing policies; tools and data on health expenditures; costing

6. **Leadership and governance:** Health sector policies; harmonization and alignment; oversight and regulation

The fact that HSS definitions encompassed 39 separate categories is indicative of the vast and complex nature of HSS policy. We found little consistency and wide variation among the definitions cited. It can be expected that taking a systems approach to health would be all-encompassing in terms of topics, but contradictions were observed in common examples of HSS. For example, we found substantial inconsistencies in the definitions of HSS used in the literature. Many authors highlighted ways that vertical programs strengthen health systems [10],[11] while others indicated that horizontal approaches better illustrated HSS [12]. Similarly, some approaches seemed to emphasize local ownership and aid effectiveness [13] while others focused on improving the efficiency of those aspects of the system that deliver their particular service [14]. Previous literature has reported a selective approach to HSS on the ground based on the priorities of different global health actors [7].

### The Need for a Consensus on Guiding Principles for HSS

The findings from our review demonstrate the diversity and inconsistencies regarding HSS definitions and approaches. The approach that an institution takes in defining HSS may influence many activities, including choices of assessment tools [15], evaluation of the impact that initiatives have on the existing health system [16], and formation of health systems training courses [17]. It is generally accepted that how a policy approach is framed conditions and constrains the range of interventions that follow. With HSS, overly specialized approaches or lack of agreement on core principles amongst different actors could limit its effectiveness. Greater consensus on guiding principles for HSS could enhance coordination and collaboration among global health actors. Given that HSS continues to gain prominence in funding, policy, and practice, we believe it is timely to begin a discourse regarding such guiding principles.

Existing HSS frameworks [9], principles [13], and strategies [14] tend to identify components of HSS or provide broad recommendations about key elements but do not provide a comprehensive list of guiding principles that are widely accepted. In contrast, our analysis focuses on underlying principles that can be a guide for specific frameworks already in use or to be developed. Therefore, despite these existing frameworks and approaches, we argue that there is value in providing overarching guiding principles for action, in contrast to recommending any specific action, which may be consistent with a particular framework or strategy. In this way, there can be broad consensus on general concepts that might direct strategies and their respective programs.

### Proposed Principles for HSS

To initiate a discourse on guiding principles on HSS and based upon our review and discussions, we propose ten key HSS principles (Box 3): holism, context, social mobilization, collaboration, capacity enhancement, efficiency, evidence-informed action, equity, financial protection, and satisfaction. Each of these principles is described briefly below.

**Box 3. Ten Health Systems Strengthening Guiding Principles**

(See http://ghsia.wordpress.com/ for discussion)

1. HOLISM
  - Consider all systems components, processes, and relationships simultaneously.
  - Include all health systems strengthening principles listed below.
2. CONTEXT
  - Consider global, national, regional, and local culture and politics.
3. SOCIAL MOBILIZATION
  - Mobilize and advocate for social and political change to strengthen health systems and address the social determinants of health.
4. COLLABORATION
  - Develop long-term, equal, and respectful partnerships between donors and recipients within the health sector and among other sectors.
  - Develop and commit to a shared vision among partners by challenging underlying beliefs and assumptions.
  - Ensure frequent communication among actors.
5. CAPACITY ENHANCEMENT
  - Enhance capacity and ownership at all levels, from individuals and households to ministries of health, including leadership, management, institutional strengthening, and problem solving.
6. EFFICIENCY
  - Train and supervise the most appropriate personnel to meet health needs.
  - Utilize appropriate technology.
  - Coordinate external aid and activities.
  - Minimize waste.
  - Allocate funds where they are needed most.
7. EVIDENCE-INFORMED ACTION
  - Strengthen structure, systems, and processes to gather, analyze, and apply data locally.
  - Make decisions, whenever possible, based on evidence.
  - Monitor progress of programs, and adjust accordingly.
  - Ensure transparency and accountability.
8. EQUITY
  - Target those who are disenfranchised.
  - Plan for equity by empowering the disenfranchised, with a particular emphasis on gender.
  - Disaggregate indicators to track disenfranchised groups.
9. FINANCIAL PROTECTION
  - Ensure that funding streams are predictable.
  - Consider insurance schemes to protect from financial catastrophes.
10. SATISFACTION
  - Respond to needs and concerns of all stakeholders.
  - Demonstrate accountability to constituents.
  - Implement and respond to feedback mechanisms measuring quality and provider/client relationships.

**1. Holism**—Health systems are continually changing and cannot be understood completely or effectively strengthened by disaggregating their different parts [2]. Strengthening one component or even several components of a health system does not necessarily strengthen the entire system; isolated actions directed to short-term goals may even weaken the overall system. Rather, global health programs should improve the overall system, with due consideration for national and local priority setting processes. Therefore, global health planners should consider the impact that their activities will have on all major components, processes, and relationships within a health system. This first principle also calls on planners to assess their activities against all of the guiding principles. For example, it is not enough to ensure donor coordination and improve supply chain management without (among other considerations) also considering the equity of those activities, the extent to which they increase local capacity, and whether they most efficiently improve the population's health.

**2. Context**—HSS activities require consideration of specific contexts and of the overall architecture of each specific system. Global health efforts involve multiple communities (e.g., host country governments, international donors, health professionals, civil society) who may have differing values and priorities about what health systems are, what they should provide, and how they should be financed and organized. These communities vary by the resources they command and the power they wield. Assumptions and beliefs about health and how services should be delivered may differ among communities as well. These important asymmetries must be understood within any given national or local context. Apart from differences amongst engaged and affected communities, there might be inherent conflicts within HSS efforts that require deliberation and informed choice. For example, some HSS efforts may reflect conflicts between equity and efficiency. Such trade-offs must be negotiated with a rigorous review of the contexts in which health systems function.

**3. Social mobilization**—HSS efforts depend considerably on social mobilization and political change. Lessons from the highly successful HIV/AIDS movement exemplifies the confluence of civil society and public health activism leading to substantial changes in global and national policies and practices. Strengthening health systems necessarily includes effective health policy reform. Many civil society organizations have successfully mobilized local groups to link communities with the formal health systems in their countries, such as Bangladesh Rehabilitation Assistance Committee's (BRAC) village organizations [18]. Some health professionals have been described as “social entrepreneurs” whose particular skill sets include the ability to initiate new civil society relationships that lead to enduring partnerships and health-promoting activities [19]. These mobilization skills should be recognized as important health system contributions to population health improvement. Training health providers should include understanding of social determinants of health and skill development in social and political advocacy to influence change in these determinants [20]. The WHO Commission on Social Determinants of Health highlighted the positive health impact of programs aimed at reducing poverty; at improving gender equality, education, nutrition, and sanitation; and at providing social protection measures to buffer market-driven inequalities [21].

**4. Collaboration**—HSS is a complex, iterative process. Global efforts at HSS require long-term partnerships with communities, and their governments, that include appreciation for the nuances of local culture and the ever-changing political and social environments. Improvements in the health status of a population often depend on policies and activities in other sectors. The influence of health professionals to promote “health in all policies” [22] rests, in part, on the development of relationships with colleagues in environment, education, economic growth, democracy and governance, media, and other sectors. Such collaborative relationships must take place on a national level among various ministries, and at district and community levels among and between providers and program planners, implementers, and users.

Effective partnerships are based on respect and dialogue that result in relationships of trust and that recognize the important, unique contributions that individuals and groups can contribute to improving health [23]. Given the inherent difference in power of donors and recipients, and the often differing underlying assumptions that determine action, it is incumbent upon donors to put in place and abide by mechanisms that foster and sustain equal partnerships. A positive health system vision of the future that is owned by all stakeholders is a powerful force for change that is just and lasting [24].

**5. Capacity enhancement**—Local capacity to detect or anticipate challenges and to solve problems is an essential component of a strong health system. Institutional capacity at the facility and regulatory levels is essential to developing a health system's ability to respond to emerging and existing health challenges within rapidly changing environments. Institutional capacity is dependent on effective leaders and management processes. A review of service delivery mechanisms suggested a strong statistical association between strong local leadership and positive health outcomes [25]. Strong management skills [26] and supervision of health providers [25] are also crucial for success. Ultimately, capacity must be enhanced at all levels from the household to health facilities to government health agencies. Particular attention should be paid to knowledge generation and application at the household and community levels through formative research and behavior change communication strategies, which can lead to stronger health systems in the long term. Such capacity enhancement is critical to enable effective community, district, and national ownership. Local ownership allows health systems to generate and manage relevant data, perform research independently, and respond rapidly to evidence by setting policy and practice priorities, and implementing effective programs.

**6. Efficiency**—Efficiency is “achieving as much of one's objectives as possible, given finite resources” [27]. Technical efficiency refers to “situations in which a good or service is produced at minimum cost” [27] and can be applied to all aspects of global health practice, from human resources to technology. Providers and practitioners at all levels should be trained, supported, and evaluated in ways that lead to maximum performance given financial inputs. Evidence strongly suggests that primary health care approaches lead to improved health outcomes [12], highlighting such approaches as foundational elements in HSS. The need for more health personnel equipped with necessary training and technology, especially public health providers [28], is well documented. Increasing evidence also suggests that community-based and household-level health promotion interventions can have a significant impact on health [29], given that from 70% to 90% of all sickness care is managed in the home [30]. Technical efficiency in global financing for HSS implies greater coordination of donor aid that is aligned with national priorities, plans, and structures, and that is predictable over time [31]. It also refers to reducing waste in the system, including redundant measurement, excessive bureaucracy, corruption, and non-productive activity.

Allocative efficiency, on the other hand, refers to whether health systems are generating the right collection of interventions required to maximize health outcomes. Programs aimed at disease and injury prevention, health promotion, reproductive health, vaccine dissemination, mental health, chronic disease, and “neglected” tropical diseases have all been argued as underfunded globally, relative to need. While efficiency remains an important principle, it must also be seen in terms of considering why some countries and sectors have scarce resources and the implications this might have for policies within and between states [32].

**7. Evidence-informed action**—Strong health systems have structures and processes in place to gather and process data and to apply that information in ways that improve performance and satisfaction. The evidence base for action at the national, regional, facility-based, and community levels is scant in low-income countries, despite the tremendous need to discern what does and does not work. Quality improvement is a process “oriented toward improving performance and using data in the process” [33] and is cyclic, iterative, and often gradual; it must be planned for. Our review and discussions suggest three primary characteristics of quality programs: 1) regular, frequent evaluations to measure impact and make changes based on that feedback; 2) flexibility and adaptation to local circumstances; and 3) accountability to constituents. Building the data infrastructure to enable transparent outcomes measurement and reporting is central to effective HSS.

**8. Equity**—Equitable health systems minimize systematic disparities that are avoidable by reasonable action [21]. Although many disparities are caused in the first instance by inequalities in social determinants of health, health systems can either exacerbate or help to reduce them, partly by how they are financed or organized. Gender equity has been identified as an especially important component of strong health systems. Three ways to meet the challenge of making health systems more equitable and capable of reducing health inequities have been proposed: first, measure and report objectives that are disaggregated to highlight disenfranchised populations, and set and report targets in terms of progress among these groups; second, modify service delivery approaches, based on experiences from innovative efforts to reach those who typically are neglected in the health system; and third, empower clients who are poor to play a more active role in the design and operation of health systems [34].

**9. Financial protection**—The financing of health systems must include mechanisms to minimize catastrophic financial impacts from ill health. Approximately 150 million people worldwide each year suffer financial catastrophe in order to pay for their health services [35]. Health financing (either through taxation or foreign aid) must be continuous and predictable, especially during financial crises when it is needed most. Experience suggests that systems with high participation in prepayment schemes avoid the impoverishing effects of out-of-pocket payments, and maximize equity [36].

**10. Satisfaction**—Finally, HSS must include attention to the satisfaction levels of all persons working within, seeking care from, or involved in programs developed by such systems. Low levels of health worker or client satisfaction, often a result of underfunded or poorly managed health systems, are associated with lower quality care and utilization rates of services and programs [37],[38]. This can imperil overall health system performance and reduce the social solidarity important to health system sustainability. A strong health system is one that demonstrates accountability to its constituents through responsiveness to their concerns and provider/client relationships they engender.

### Toward a Consensus

Our collective experience, discussion with experts throughout the world, and review of the literature demonstrates the need for a consensus on guiding principles for HSS. The principles that we list have already been applied to differing degrees in the policies and practices of many global health initiatives and institutions. However, to our knowledge, there has been no unified application of a set of HSS guiding principles to facilitate communication and collaboration between donors and recipient states and communities. We offer the principles above as a contribution to the ongoing discussion and debate around the language and practice of HSS. Our principles need to be field-tested and evaluated in an array of settings, such as in health systems impact assessments [39], programmatic interventions, and research activities with support from a variety of major global health stakeholders. We invite global health leaders and planners to scrutinize and counter these principles, and we hope that such a discussion will establish a common set of principles that will serve as the foundation for future HSS discussions and strategies.

## Supporting Information

Text S1 Methodology for Comprehensive, Systematic Review of Current HSS Definitions
(0.03 MB DOC)

Text S2 Keywords
(0.03 MB DOC)

Text S3 Conferences Where the Health Sytems Strengthening Guiding Principles Were Discussed and Debated
(0.03 MB DOC)
