# Supplementary material for: Toward a Consensus on Guiding Principles for Health Systems Strengthening
Source: PLoS Med. 2010 Dec 21;7(12):e1000385. doi: 10.1371/journal.pmed.1000385 (PMC3006350; doi:10.1371/journal.pmed.1000385)
Supplement: Text S1 — Methodology for Comprehensive, Systematic Review of Current HSS Definitions (0.03 MB DOC) [file pmed.1000385.s001.doc]

**Text S1: Methodology for Comprehensive, Systematic Review of Current HSS Definitions**

*Sources of Data*

We conducted a systematic literature review that included both peer-reviewed and grey literature. We searched PubMed, Google Scholar, and Scopus for peer-reviewed literature published from 2000-2009. For grey literature sources, we employed a Google search, peer consultation, and individual judgment, which included websites, conference proceedings, interviews, textbooks, and white papers.

Search Terms and Inclusion Criteria

The search term “health system(s) strengthening” was used to identify relevant data.

Two independent researchers (VM and AO) performed a full-text review of all identified literature to determine if they met all of the following inclusion criteria:

1. Includes a definition, explanation, or example of strengthening or improving health systems
2. Relevant to low- or middle-income country context
3. English language

Data Collection

Following the independent inclusion process by both reviewers, we merged results and resolved conflicts through consensus, as seen in the diagram below.

Figure 1: Flow diagram of inclusion process

Records identified through database searching

(n = 871)

**Identification**

Additional records identified through other sources

(n = 56)

Records after duplicates removed

(n =633)

**Screening**

**Included**

**Eligibility**

Full-text articles assessed for eligibility

(n =633)

Full-text articles not meeting inclusion criteria

(n =296)

Studies included in synthesis and analysis

(n =337)

*Adapted from:*  Moher D, Liberati A, Tetzlaff J, Altman DG, The PRISMA Group (2009). *P*referred *R*eporting *I*tems for *S*ystematic

Reviews and *M*eta-*A*nalyses: The PRISMA Statement. PLoS Med 6(6): e1000097. doi:10.1371/journal.pmed1000097
