# Supplementary material for: Toward a Consensus on Guiding Principles for Health Systems Strengthening
Source: PLoS Med. 2010 Dec 21;7(12):e1000385. doi: 10.1371/journal.pmed.1000385 (PMC3006350; doi:10.1371/journal.pmed.1000385)
Supplement: Text S2 — Keywords (0.03 MB DOC) [file pmed.1000385.s002.doc]

# **Text S2**

Keywords for defining HSS (***Further clarification on key words in italics*)**

- Health service delivery and packages/delivery models; infrastructure; demand for care
- Management/administrative functions
- *S*afety & quality *(related to service delivery but NOT medical products)*
- Health workforce related national policies and investment plans; norms, standards and training *(any sort of investment, training or emphasis on human resources with respect to doctors, nurses, community health workers, etc.)*
- Facility and population based information & surveillance systems; global standards, tools
- Norms, standards for medical products, quality and policies; reliable procurement *(anything related to drugs or medical technologies including logistics surrounding the management of these products)*
- National health financing policies/insurance coverage; tools and data on health expenditures/costing
- Pay for performance/incentives *(payment based on results/quality)*
- Ownership; harmonization and alignment in governance; oversight and regulation; accountability; leadership development
- Public-private partnerships /collaboration and integration of stakeholders *(the inclusion of NGOs, civil society organizations, etc. with governments in a partnership)*
- Mention of all 6 WHO building blocks
- Any initiatives that aim to better health or the health system *(applies if the definition does not get more specific than this)*
- Access to care
- Civil society/NGO *activities (emphasis on fostering programs with these groups but in a way not necessarily related to leadership partnerships or contracts with the government)*
- Capacity building *(applies if the definition does not get more specific than this)*
- Primary health care *(treat as if separate from maternal or child health)*
- Maternal health
- Child or under-5 health
- Context-specific: country driven; relevant to disease burden *(when the interventions relevant to the environment they are being implemented in; this is not the same as evidence-based interventions)*
- Involvement of all stakeholders *(ensuring all stakeholders have a role, but not necessarily in a partnership)*
- Results oriented *(programming is based on getting results and meeting goals)*
- Sector-wide approach (SWAp) *(to be tagged ONLY when explicitly mentioned exactly as SWAP)*
- Synergistic activities *(leverages synergies that arise when multiple issues are addressed)*
- Vertical approach: disease or service specific activities
- Horizontal approach: non-disease or non-service specific activities
- Diagonal approach: combination of vertical and horizontal
- Community centered/empowerment *(a focus on local community)*
- Scale-up district/local health projects
- Private-sector contracting *(policies that focus on the government granting contracts to the private sector or NGOs for health services it would traditionally provide)*
- Decentralization *(policies that move power or authority from central government control to more local government control; a more equal distribution of power)*
- Public-sector focus *(focus on government activities)*
- Health systems research
- Sustainable
- Equity agenda *(related to programming that addresses inequities and barriers for poor or marginalized groups)*
- Evidence-based practice *(programs or policies that are based on evidence in the literature or past efforts; NOT the same as programming that is context specific)*
- Efficiency
- Social determinants of health *(related to the concept of social capital and how social constructs like race, religion, socioeconomic status etc. can affect health)*
- Monitoring and evaluation *(efforts to regularly monitor and evaluation interventions)*
- Other (specify)
