# Supplementary material for: Toward a Consensus on Guiding Principles for Health Systems Strengthening
Source: PLoS Med. 2010 Dec 21;7(12):e1000385. doi: 10.1371/journal.pmed.1000385 (PMC3006350; doi:10.1371/journal.pmed.1000385)
Supplement: Text S3 — Conferences Where the Health Sytems Strengthening Guiding Principles Were Discussed and Debated (0.03 MB DOC) [file pmed.1000385.s003.doc]

**Text S3: Conferences Where the Health Sytems Strengthening Guiding Principles Were Discussed and Debated**

1. **CORE Group 2009 Fall Community Health Network Meeting**, October 8, 2009. Washington, DC, USA. R. Chad Swanson presented “Consensus Statement on Health Systems Strengthening.”
2. **Health Impact Assessment Conference**, October 15, 2009. Rotterdam, Netherlands. R. Chad Swanson presented “Health Systems Impact Assessments.”
3. **Global Forum for Health Research 2009**, November 14-20, 2009. Havana, Cuba. R. Chad Swanson led discussions in the marketplace on “Consensus Statement on Health Systems Strengthening.”
4. **Global Family Health Conference**, April 13, 2010. Provo, Utah, USA. R. Chad Swanson presented on “Health Systems: Strengthening toward a Consensus and a Social Movement.”
5. **University of Utah Global Health Alliance Global Health Conference**, May 7, 2010. Salt Lake City, Utah, USA. R. Chad Swanson presented on "Health Systems Strengthening in Global Health:  What Does it Mean, and Are We Doing it?”
6. **Global Health Council 2010 Conference**, June 14-June 13. Washington DC, USA. Several co-authors discussed the principles in a presentation, and various informal discussions.
